# Malum Perforans in Diabetes

**Published:** 1913-03

**Authors:** 


Malum Perforans in Diabetes. John T. Sample and W. L. Gorham, *Johns Hopkins Hosp. Bull.,* Jan., 1913. Seven cases of perforating ulcer of the toes, foot, etc., are reported. Among 687 cases of diabetes in literature, 19 showed ulcers—2.7 per cent. Gascuel, conversely, found among 91 cases of malum perforans, that 14 were diabetics—15 per cent. Various plausible theories are discussed with negative conclusions In no case of diabetic malum perforans was neuritis demonstrated. The mechanic theory of pressure is confirmed by the fact that the majority of cases are in males who would be supposed to use the feet more (but not to subject them to more pressure) and by observations of cure on allowing rest. On the other hand, some cases are not thus relieved and the ulcer may occur on the hand. Endarteritis is either absent, or developes late. The toxaemic theories (lessened resistance on account of hyperglycaemia or of toxins in the stricter sense) are negatived by the fact that malum perforans does not coincide except occasionally with gangrene, carbuncles, etc., that it is recorded in only one case of severe glycosuria and that it occurs mainly in mild cases, without acetone bodies. The knee jerk is absent in about half of the cases.

